# Effect of Pre-IVM Duration with cAMP Modulators on the Production of Cloned Equine Embryos and Foals

**DOI:** 10.3390/ani15131961

**Published:** 2025-07-03

**Authors:** Jenin V. Cortez, Kylie Hardwicke, Carlos E. Méndez-Calderón, Christopher G. Grupen

**Affiliations:** 1 Sydney School of Veterinary Science, Faculty of Science, The University of Sydney, Camden, NSW 2570, Australia; 2 Catalina Equine Reproduction Centre, North Richmond, NSW 2754, Australia

**Keywords:** in vitro maturation (IVM), biphasic IVM, simulated physiological oocyte maturation (SPOM), somatic cell nuclear transfer (SCNT), blastocyst, embryo transfer, horse

## Abstract

The asynchrony of cytoplasmic and nuclear maturation in cumulus–oocyte complexes (COCs) due to prematurely declining concentrations of cyclic adenosine monophosphate (cAMP) has been shown to result in reduced oocyte developmental competence. The objective of this study was to evaluate the effect of pre-IVM treatment with cAMP modulators for different durations on the developmental potential of equine oocytes used for cloned embryo production. Collected COCs were transferred to cryovials filled with transport medium at 20–22 °C. Within the cryovials, the COCs were either untreated (Control) for 18 h or treated with 50 µM forskolin and 100 µM 3-isobutyl-1-methylxanthine for the first 4 h (Pre-IVM 4 h) or the entire 18 h (Pre-IVM 18 h). Oocytes were then transferred to maturation medium and incubated for a further 22–24 h at 38.5 °C in 5% CO_2_ in air. Somatic cell nuclear transfer embryos were then produced using the meiotically mature oocytes and donor cells from six different fibroblast cell lines. The rates of maturation and embryo development did not differ significantly between the groups, though blastocyst formation tended to be inferior in the Pre-IVM 4 h group compared with the Control group (*p* = 0.06). Of 67 blastocysts produced, 23 were transferred to recipient mares on Day 4 or 5 post-ovulation. Regarding the pregnancy outcomes, no significant differences were found between the groups, and four viable foals were born, each derived from a different donor cell line. The findings expand on those from previous evaluations of this biphasic IVM system, and indicate that the cAMP-modulating treatments exert limited effects under the pre-IVM conditions used here.

## Simple Summary

The efficiency of equine embryo in vitro production is mainly limited by the capacity of current in vitro maturation (IVM) systems to support the acquisition of oocyte developmental competence. Oocyte quality may be improved by better coordinating nuclear and cytoplasmic maturation using pre-IVM treatments that modulate cAMP levels. The aim of this study was to evaluate the effect of pre-IVM treatment with cAMP modulators for short and long durations on equine oocyte quality. Following maturation with or without the pre-IVM treatments, the oocytes were used to produce cloned embryos and early development was assessed. Additionally, cohorts of blastocysts were transferred to recipient mares and the resulting pregnancies were monitored. The in vitro development of embryos did not differ significantly between the groups, though blastocyst formation tended to be inferior when the oocytes were subjected to the short pre-IVM. The in vivo development of transferred blastocysts was not adversely impacted by the pre-IVM treatments, with pregnancies established and foals born in all groups. While the use of cAMP modulators in this biphasic IVM system supported successful outcomes, it did not enhance the production of cloned equine embryos and foals.

## 1. Introduction

In horses, assisted reproductive technologies (ARTs) are constantly being refined to enhance or extend the reproductive potential of valuable animals. While oocyte in vitro maturation (IVM) can successfully generate viable embryos after transfer [1], a major focus in recent years has been on improving oocyte IVM systems. This is particularly important when oocytes are used to produce cloned embryos, as the inefficiencies inherent in the somatic cell nuclear transfer (SCNT) procedure necessitate a large number of oocytes as starting material [2]. In vivo, oocyte maturation is regulated by signals from the somatic compartment of the follicles, including granulosa and cumulus cells, which coordinate the acquisition of developmental competence, such that the oocyte cytoplasmic components correctly direct the intricate events following fertilization and oocyte activation [3,4].

The development of IVM for equine embryo production has been slower compared to other domestic animals due to lower success rates and the constraints in obtaining immature oocytes [4]. Asynchrony of nuclear and cytoplasmic maturation results when immature oocytes are removed from antral follicles, contributing to the poor developmental potential of IVM oocytes [5]. A commonly used maturational synchronizing strategy is to simulate the conditions that maintain oocyte meiotic arrest at prophase I stage by elevating the intra-oocyte concentrations of cAMP, a key regulator of meiotic progression [6,7]. Transient exposure of oocytes to cAMP-elevating agents inhibits the degradation of germinal vesicles through the activation of protein kinase A and prevents the spontaneous resumption of meiosis, thereby reducing the asynchrony of cytoplasmic and nuclear maturation [8,9].

The cAMP modulating treatments used in the so called Simulated Physiological Oocyte Maturation (SPOM) system have been shown to exert beneficial effects on the in vitro production of embryos in several species, including cattle [10,11,12], goats [13], horses [14], and mice [10,15]. However, the effectiveness of the SPOM system remains contentious, owing to the inconsistent results achieved by different research groups [16]. This pre-IVM approach uses a combination of intra-oocyte cAMP modulators, specifically forskolin (FSK), which activates adenylate cyclase and increases cAMP synthesis, and isobutyl-1-methylxanthine (IBMX), which acts as a phosphodiesterase (PDE) inhibitor and prevents cAMP catabolism. The combined activities stimulate greater elevation of cAMP levels than either activity alone and supposedly maintain oocyte meiotic arrest in a way that more closely mimics that in an in vivo environment [10]. Interestingly, the duration of the SPOM pre-IVM incubation varies considerably between studies, with an overnight duration being used successfully in horses [14], and shorter durations (2–6 h) being beneficial in cattle [11,12]. Overnight pre-IVM treatment of equine oocytes is logistically convenient, as this coincides with the period typically needed to transport them from the site of collection to the laboratory for subsequent maturation and embryo production.

The objective of this study was to evaluate the effectiveness of the pre-IVM treatment (FSK and IBMX combined) applied for different durations (4 and 18 h) on the developmental competence of equine oocytes recovered from slaughterhouse-sourced ovaries. After IVM (with or without the pre-IVM treatments), matured oocytes were used to produce cloned embryos by SCNT, and the rates of maturation, couplet fusion, embryonic cleavage, and blastocyst formation were assessed. Following the transfer of blastocysts to recipient mares, pregnancy outcomes were also monitored.

## 2. Materials and Methods

### 2.1. Mares

This study was conducted at the Catalina Equine Reproduction Centre located in North Richmond, NSW, Australia. A total of 23 standardbred mares, ranging in age from 3 to 15 years, served as recipients for embryo transfer. All procedures were performed with the informed consent from the animals’ owners and in compliance with the NSW Animal Research Act (1985), which incorporates the Australian Code of Practice for the Care and Use of Animals for Scientific Purposes [17].

### 2.2. Chemicals and Media

Unless specified otherwise, all chemicals and reagents were obtained from Sigma-Aldrich (Bayswater, VIC, Australia). Hepes-buffered Synthetic Oviductal Fluid (H-SOF) [18] supplemented with 10% fetal calf serum (FCS; AU-FBS/PG; Cellsera, Rutherford, NSW, Australia) was used when procedures were conducted outside of the CO_2_ incubator. The transport medium used to hold oocytes prior to IVM (including the pre-IVM treatments) was a 1:1 mixture of Dulbecco’s Modified Eagle’s Medium/Nutrient Mixture F-12 Ham (DMEM/F-12; D8437) and Medium 199 (M3769) supplemented with 10% FCS. The oocyte maturation medium and the couplet fusion medium were prepared as previously described [19].

### 2.3. Collection of Immature Oocytes

Mare ovaries were obtained at a slaughterhouse and processed within 2 h. The process of cumulus–oocyte complex (COC) recovery has been described in detail previously [19]. Briefly, using an 18G needle, antral follicles < 30 mm in diameter were aspirated, and, using a bone curette, the follicle walls were scraped extensively. Following transfer of follicular material to H-SOF warmed at 37 °C within a 100 mm Petri dish, COCs were selected and washed three times with H-SOF. A total of 715 COCs were collected in 6 replicates (mean of 119.2 COCs per replicate; range of 82 to 146). In each replicate, the COCs were allocated to one of three pre-IVM treatment groups such that each group was transferred to a separate 1.5 mL cryovial containing transport medium at 20–22 °C. The cryovials were securely capped and kept within a polystyrene container at 20–22 °C for overnight transport to the laboratory and before IVM. Due to the logistics of SCNT processing after oocyte IVM, the allocation of COCs to the cryovials of transport medium, the transfer of COCs to IVM medium 18 h later, and the processing of oocytes for SCNT were staggered in the same order to achieve consistent timing. The three cryovials contained either (i) non-supplemented transport medium for the entire 18 h duration (Control), (ii) transport medium supplemented with 50 µM forskolin (FSK) and 100 µM 3-isobutyl-1-methylxanthine (IBMX) for the first 4 h and then non-supplemented transport medium for the next 14 h (Pre-IVM 4 h), and (iii) transport medium supplemented with FSK and IBMX for the entire 18 h duration (Pre-IVM 18 h). The concentrations of FSK and IBMX were selected based on the findings of Metcalf and co-workers [14,20].

### 2.4. Oocyte In Vitro Maturation (IVM)

At the end of the 18 h transport and holding period, the COCs of each group were washed with H-SOF three times and placed into wells of 4-well dishes (144444; Nunc, Roskilde, Denmark) containing 500 µL of pre-equilibrated maturation medium (20–50 COCs per well). The COCs were incubated for 22–24 h at 38.5 °C in a humidified atmosphere of 5% CO_2_ in air.

### 2.5. Somatic Cell Nuclear Transfer (SCNT)

A total of 6 different fibroblast cell lines were derived from the subcutaneous tissue of 6 adult horses (one male warmblood, and two male and three female polo ponies). A different cell line (denoted as #01 to #06) was used in each of the 6 replicates. DMEM/F-12 medium supplemented with 1 mM glutamine, 0.2 mM pyruvate, 10 ng/mL EGF, and 10% FCS was used to culture the fibroblast cells. After plating and initial expansion, the cells were subcultured twice before freezing at −80 °C in culture medium supplemented with 10% dimethyl sulfoxide (DMSO) and storing in liquid nitrogen. After thawing the cells, they were cultured for at least two days. To arrest the cell cycle, the cells were grown to confluence at least 24 h before use as donor cells. About 10 min prior to use for SCNT, a suspension of the donor cells was prepared at room temperature (RT) by trypsinizing, washing, and transferring them to H-SOF supplemented with 2% FCS. Fresh donor cells were prepared at the start of the SCNT procedure for each group of oocytes.

After IVM, the oocytes were denuded of cumulus cells by gentle pipetting with 1 mg/mL hyaluronidase in H-SOF. Each metaphase-II-stage oocyte was visualized using an inverted microscope equipped with Hoffman modulation contrast optics and enucleated using a micropipette attached to a Piezo drill (PMAS-CT150; Prime Tech Ltd., Ibaraki, Japan). Oocytes were considered to be successfully enucleated when the birefringence properties of the meiotic spindle were observed upon aspiration. Each resulting cytoplast then had a donor cell placed in the perivitelline space. A total of 308 couplets were constructed in 6 replicates (mean of 51.3 couplets per replicate; range of 33 to 65). The couplets were incubated for 1 h in H-SOF at 38 °C, before being transferred to fusion medium placed between the electrodes (0.5 mm apart) of a fusion chamber slide. A direct current (DC) pulse of 2.2 kV/cm strength and 15 µsec duration was applied to the couplets immediately, after which they were washed several times and then incubated for 2 h in embryo culture medium at 38.5 °C. Subsequently, the fused couplets were activated by exposing them to 5 µM ionomycin in H-SOF for precisely 5 min, washed several times in H-SOF, and incubated for 4 h in embryo culture medium containing 1 mM 6-dimethylaminopurine and 5 µg/mL cycloheximide. After further washes, the SCNT embryos were transferred to 10 µL droplets of embryo culture medium (maximum of 7 embryos in each droplet), and incubated in a humidified atmosphere of 5% CO_2_, 5% O_2_ and 90% N_2_ at 38.5 °C. The SCNT embryos were transferred every 2 days to droplets of fresh embryo culture medium [19]. At the first change of embryo culture medium, cleavage was assessed. Blastocyst development was evaluated at Days 7 and 8, using well-described morphological features to classify blastocyst quality [21].

### 2.6. Blastocyst Vitrification and Thawing

Blastocysts were vitrified using the Cryotop method following the manufacturer’s directions (Kitazato BioPharma, Shizuoka, Japan). In summary, 300 µL of equilibration solution (ES) and vitrification solution 1 (VS1) were deposited in separate wells of a 4-well dish at RT. Embryos were transferred to the ES drop for 12–15 min and a cycle of contraction (dehydration) and re-expansion (ES infiltration) was observed. Then, the equilibrated embryos were transferred to VS1 for 1 min, after which each embryo was placed with a minimal amount of medium on the thin polypropylene strip of the Cryotop, and the device was immediately immersed vertically in liquid nitrogen. To thaw, the Cryotop was directly immersed in 1 mL of prewarmed (37 °C) thawing solution (TS) for 1 min. Embryos thawed in TS were gently placed at the bottom of a 300 µL drop of dilution solution (DS) for a gradual shift from TS to DS over 3 min at RT. The embryos were drawn up into a pipette tip with a 2 mm column of DS and then carefully placed at the base of a 300 µL drop of warming solution 1 (WS1), for a gradual shift from DS to WS1 over 5 min at RT. The embryos were then washed twice for 1 min in warming solution 2 (WS2). Finally, the embryos were transferred to equilibrated and warmed (38.5 °C) embryo culture media.

### 2.7. Embryo Transfer (ET)

Non-surgical embryo transfers were performed in 23 standardbred mares as described previously [19]. For each embryo that was transferred, the size of the somatic cell nucleus donor animal was considered to select an appropriately sized recipient mare able to carry the pregnancy to term. Within 30 min of warming, blastocysts were transferred trans-cervically to recipients on Day 4 or 5 post-ovulation. Pregnancies were diagnosed by transrectal ultrasonography on Days 14 (Day 9 after ET), 45 and 90 of gestation. During pregnancy, fetal movements, placental quality, and heart rate were monitored. Additional scans were subsequently performed once a month to monitor the progress of ongoing pregnancies.

### 2.8. Statistical Analysis

Analyses of the data were performed using Genstat for Windows 22nd Edition statistical software (Version 22.1.0.195; VSN International, Hemel Hempstead, UK). The proportional SCNT embryo production data (oocytes matured, couplets fused, embryos cleaved, and blastocysts formed) were subjected to logistic regression analysis using the logit transformation, with treatment group and cell line as factors. When no significant differences were detected, the variance between groups was determined using ANOVA and Fisher’s unprotected pairwise comparison. The pregnancy and foaling data were analyzed using chi-square tests to evaluate the null hypothesis that there was no difference between the groups. A *p* value less than 0.05 was considered to be statistically significant.

## 3. Results

### 3.1. In Vitro Development of SCNT Embryos

#### 3.1.1. Effect of cAMP-Modulating Pre-IVM Treatments

**Figure 1:**
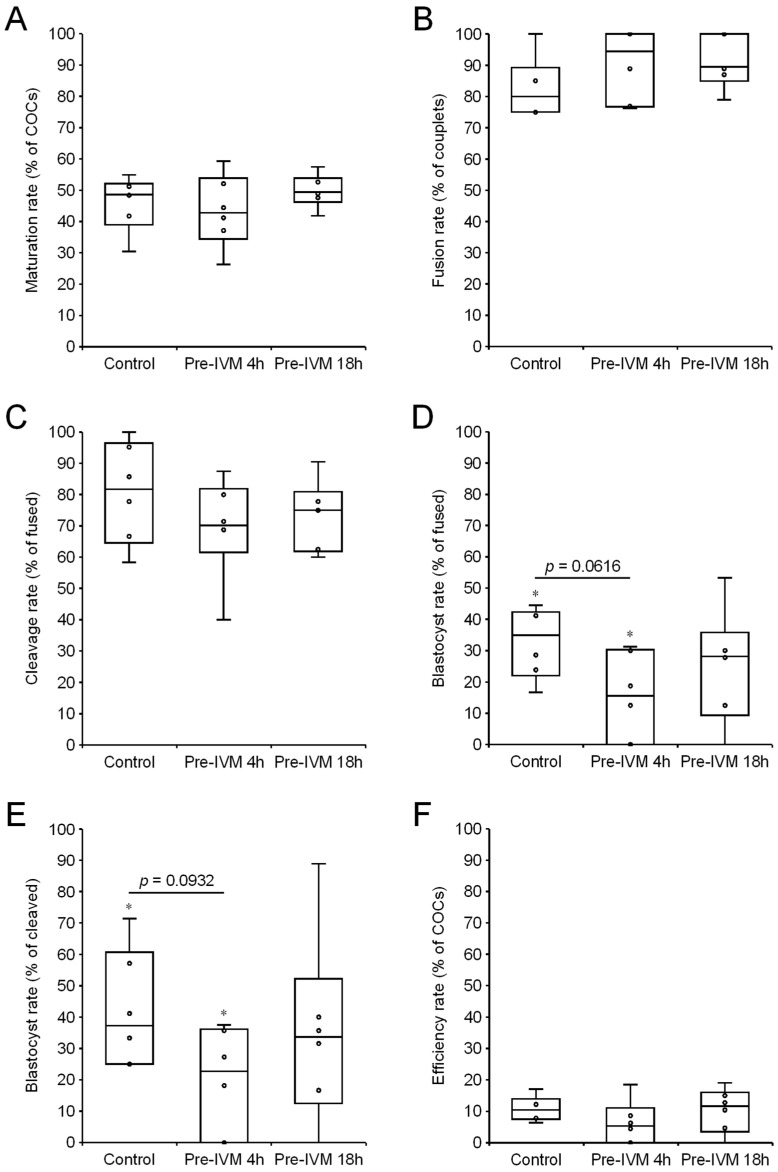
Effect of the cAMP-modulating pre-IVM treatments on SCNT embryo production (Control, Pre-IVM 4 h, and Pre-IVM 18 h groups). (**A**) The percentage of oocytes that matured after an additional 22–24 h of IVM, as determined by the presence of a first polar body. (**B**) The percentage of constructed couplets that fused. (**C**) The percentage of fused couplets that cleaved after 48 h of embryo culture. (**D**) The percentage of fused couplets that formed blastocysts. (**E**) The percentage of cleaved embryos that formed blastocysts. (**F**) The overall cloning efficiency rate, expressed as the percentage of cumulus–oocyte complexes (COCs) that resulted in blastocysts. Values are presented as the mean ± s.e.m. No significant differences were detected between the groups (*p* > 0.05). Values labeled with an asterisk tend to differ (*p* < 0.1).

The effects of the cAMP-modulating pre-IVM treatments on the in vitro production and development of SCNT embryos are shown in Figure 1. In the six replicates, a total of 266 oocytes were allocated to the Control group (mean of 44.3 oocytes per replicate), a total of 191 oocytes were allocated to the Pre-IVM 4 h group (mean of 31.8 oocytes per replicate), and a total of 258 oocytes were allocated to the Pre-IVM 18 h group (mean of 43.0 oocytes per replicate). The rates of maturation, couplet fusion, embryonic cleavage and overall cloning efficiency did not differ between the groups (*p* > 0.05). However, the rates of blastocyst formation, as proportions of fused couplets and cleaved embryos, tended to be lower in the Pre-IVM 4 h group than in the Control group (*p* < 0.1). The blastocyst rates of the Pre-IVM 18 h group did not differ from those of the Control and Pre-IVM 4 h groups (*p* > 0.05). Using untreated Control oocytes, blastocysts were produced in all six replicates (mean of 4.5 blastocysts per replicate). Conversely, blastocysts were produced in five of the six replicates using the Pre-IVM 18 h treated oocytes (mean of 4.5 blastocysts per replicate), and in four of the six replicates using the Pre-IVM 4 h treated oocytes (mean of 2.2 blastocysts per replicate).

#### 3.1.2. Effect of Donor Cell Lines

**Figure 2:**
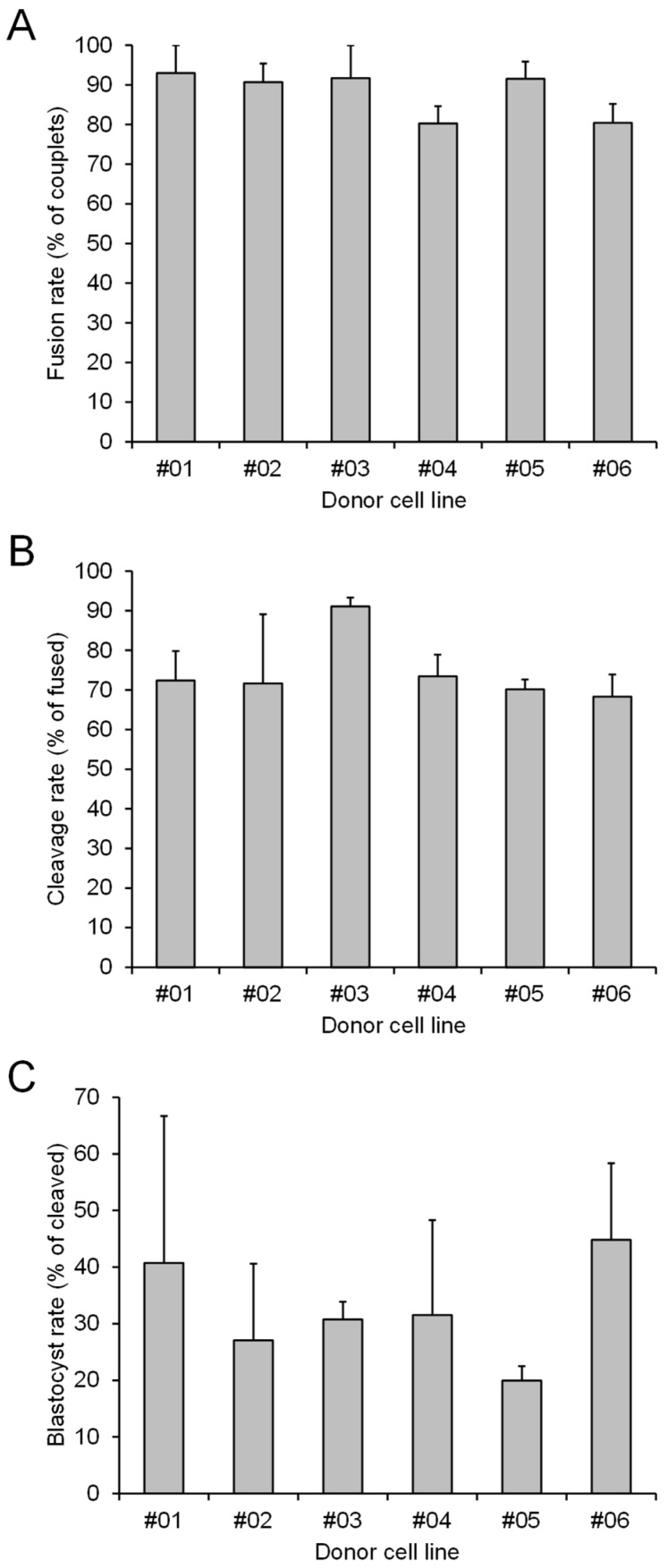
Effect of the donor cell lines (denoted as #01 to #06) on SCNT embryo production. (**A**) The percentage of constructed couplets that fused. (**B**) The percentage of fused couplets that cleaved. (**C**) The percentage of cleaved embryos that formed blastocysts. Values are presented as the mean ± s.e.m. No significant differences were detected between the donor cell lines (*p* > 0.05).

A total of six different fibroblast cell lines, generated from separate skin biopsies, were used to provide the donor nuclei used to produce the SCNT embryos. Each cell line was used in all three experimental groups (Control, Pre-IVM 4 h, and Pre-IVM 18 h), such that a different cell line was used in each of the six replicates. There was no effect of donor cell line on the rates of couplet fusion, embryonic cleavage, or development to the blastocyst stage (*p* > 0.05; Figure 2). There was no significant interaction between treatment and cell line, and a mean of 10.8 blastocysts was produced for each cell line (range of 7 to 16).

### 3.2. Pregnancy Outcomes After ET

#### 3.2.1. Effect of cAMP-Modulating Pre-IVM Treatments

Of the 67 blastocysts produced and vitrified, a total of 23 blastocysts were warmed and transferred (4, 2, and 17 for the Control, Pre-IVM 4 h, and Pre-IVM 18 h groups, respectively). The results of pregnancy diagnosis after transfer of the SCNT embryos to recipient mares are shown in Table 1. As determined by ultrasonographic detection of an embryonic vesicle, 10 were viable on Day 14. Although the proportions of mares in which embryonic vesicles were detected appeared to vary between the groups, the Day 14 pregnancy rates did not differ significantly (25%, 50%, and 47% for the Control, Pre-IVM 4 h, and Pre-IVM 18 h groups, respectively; *p* > 0.05).

At Day 45 of pregnancy, the conceptuses of the Control and Pre-IVM 4 h groups remained viable, while pregnancy losses occurred in the Pre-IVM 18 h group, and two conceptuses remained viable. Beyond Day 45 of gestation, no pregnancy loss occurred in any of the groups. A total of four foals were born (one, one, and two in the Control, Pre-IVM 4 h, and Pre-IVM 18 h groups, respectively). Two of the foals were born normal and healthy, and the other two were declared healthy after successful treatment of minor forelimb and umbilical problems.

#### 3.2.2. Effect of Donor Cell Lines

The pregnancy results obtained for the different cell lines are shown in Table 2. A total of 23 blastocysts produced from four of the six cell lines were transferred to 23 recipient mares, and 10 of the transferred blastocysts formed embryonic vesicles at Day 14 of pregnancy. All of the resulting Day 45 conceptuses completed full-term development, such that four foals were born, one derived from each of the four donor cell lines involved.

#### 3.2.3. Effect of Embryo Grade

The effect of the quality of the transferred embryo (Grade 1 vs. Grade 2) on pregnancy outcome is shown in Table 3. A total of 16 Grade 1 blastocysts and 7 Grade 2 blastocysts were transferred to the recipient mares. While pregnancies were detected at Day 14 for both embryo grades, only Grade 1 blastocysts established ongoing pregnancies and generated viable births (Grade 1: 4/16; Grade 2: 0/7). However, the apparent difference was not statistically significant (*p* > 0.05).

#### 3.2.4. Effect of Recipient Mare’s Day Post-Ovulation

The effect of the recipient mare’s day post-ovulation (Day 4 vs. Day 5) at ET was also analyzed. As shown in Table 4, the transfer of embryos to recipients on Days 4 and 5 after ovulation resulted in similar Day 14 pregnancy rates. While more pregnancy losses occurred in the Day 5 post-ovulation group compared with the Day 4 post-ovulation group, the pregnancy rates at Days 45 and 90 and the foaling rates did not differ significantly (2/7 and 2/16, respectively).

## 4. Discussion

The results of this study show that the developmental competence of equine oocytes used to produce embryos by somatic cell nuclear transfer (SCNT) was not improved following pre-IVM treatment with the cAMP modulators forskolin (FSK) and 3-isobutyl-1-methylxanthine (IBMX), a previously described biphasic IVM system referred to as Simulated Physiological Oocyte Maturation (SPOM) [10,16]. A total of 715 oocytes were recovered from slaughterhouse-sourced ovaries in six replicates, and a total of 67 blastocysts were produced. While no significant differences between the treatments and Control groups were found, the blastocyst formation rates tended to be lower in the Pre-IVM 4 h group than in the Control group. Within the constraints of a commercial equine cloning program, 23 blastocysts were transferred to recipient mares, resulting in the birth of four healthy foals derived from four different donor cell lines. There were no significant differences in pregnancy outcomes between the treatments and Control groups, demonstrating for the first time that pre-IVM exposure of equine oocytes to FSK and IBMX does not prevent full-term development of SCNT embryos. These findings expand the evaluation of the SPOM system in equine oocytes.

The developmental competence of in vitro-matured oocytes is compromised due to the initiation of spontaneous meiotic resumption, which occurs in response to a decrease in the cAMP levels responsible for maintaining meiotic arrest [3,22]. Adequate levels of cAMP in oocytes are generated and maintained by granulosa cells, cumulus cells, and metabolites of the follicular compartment to ensure that meiosis resumes in an orchestrated manner [23]. By disrupting the oocyte–follicle connection, the intra-oocyte cAMP levels decrease, such that the resumption of meiosis occurs in a more rapid and uncontrolled manner [24]. In horses, the problem is exacerbated because the procedures used to recover oocytes via ovum pick up (OPU) or from slaughterhouse ovaries are more challenging than those used in other species [25,26]. Similar to the approaches described previously in horses and other species [16,20], the rationale for the pre-IVM treatments used here was to maintain the intra-oocyte cAMP concentrations immediately after collection, thus avoiding spontaneous meiotic resumption.

Research carried out in equine oocytes using treatments to inhibit meiosis is limited. The study by Choi et al. [27] was one of the first to report the manipulation of meiotic resumption in this species using roscovitine, which is an analog of purine that specifically inhibits M-phase-promoting factor activity. The addition of roscovitine to pre-IVM medium for 16–18 h effectively maintained oocytes at the germinal vesicle stage, but their developmental competence after subsequent IVM and intracytoplasmic sperm injection (ICSI) was not enhanced [27]. Conversely, Metcalf et al. [14,20] found that the rates of maturation and blastocyst development were increased, compared with the Control, when equine oocytes were held overnight in medium with 50 µM FSK and 100 µM IBMX (i.e., the SPOM system) [14,20]. Using the same concentrations of FSK and IBMX as those used by Metcalf et al. [14], which were found to be optimal for equine oocytes in a pilot study [20], the results of the present study did not show any significant differences in the rates of maturation, couplet fusion, cleavage, and blastocyst formation between the pre-IVM and Control groups. The inconsistent results between the studies may be due to the different media used or the possibility that supplementation with serum may have interfered with the pre-IVM treatments [16]. Another major difference between the studies is that Metcalf et al. [14,20] produced ICSI embryos using oocytes harvested from live mares by OPU, whereas here we produced SCNT embryos using oocytes recovered from slaughterhouse-sourced ovaries. In a previous study we found that oocyte source (OPU-derived vs. abattoir-derived) significantly impacted the development of SCNT embryos to the blastocyst stage [19].

The SPOM system has been applied to embryo production in numerous species, including cows [10,11,28,29,30,31], mice [10,15,32], sheep [7,33], goats [13], horses [14], and cats [34]. The effectiveness of the SPOM treatment has not been consistent, with only 34.7% (8/23) of studies achieving an improvement in blastocyst production [16]. Further, in those studies conducted in cattle, only 25% (4/16) of them succeeded in improving blastocyst production [16]. Differences between studies in the composition of the pre-IVM base medium and the supplements used suggest there are complex interactions with factors in the media that influence the effectiveness of the cAMP modulators [16].

A major variable of the SPOM system is the duration of the FSK and IBMX treatment. In horses, oocytes are often collected at a site distant to the laboratory, such that transportation overnight, as was the case in this study, is necessary. Hence, for the Pre-IVM 18 h group, the oocytes were kept in transport medium supplemented with FSK and IBMX for the entire transportation period, whereas for the Pre-IVM 4 h group, the oocytes were first held in transport medium supplemented with FSK and IBMX for 4 h before being transferred and kept in transport media without the cAMP modulators for the remaining 14 h. The oocytes of the Control group were kept in transport medium without the cAMP modulators for 18 h. It is unclear why the Pre-IVM 4 h treated oocytes tended to have poorer developmental potential compared with the untreated Control oocytes. A possible explanation is that the additional handling and medium change had a negative influence. In the only other horse studies of the SPOM system, an overnight pre-IVM duration was used with some success [14,20], while in cattle studies of the SPOM system, using pre-IVM durations of 2 and 6 h achieved positive results [10,12,35,36]. Further studies are needed to determine whether an alternative pre-IVM duration may be optimal. A key feature of equine oocyte maturation is the ability to hold immature oocytes at room temperature for 18 h, which appears to promote chromatin condensation at the germinal vesicle stage without reducing developmental competence [37,38]. As the modulation of cAMP levels by the SPOM system has been carried out at 37–38.5 °C in other species [10], an influence of the pre-IVM incubation temperature on the treatments cannot be ruled out.

The blastocysts produced were vitrified and then transferred into previously synchronized recipient mares. Due to the commercial imperative to obtain foals from particular donor cell lines, a similar number of blastocysts from each group could not be transferred. Pregnancy rates on Day 14 of gestation did not differ among the groups. Of the embryonic vesicles that developed, 40% (4/10) were viable conceptuses on Day 45 of gestation. Pregnancy loss and compromised neonatal health associated with equine cloning have been attributed to defective epigenetic reprogramming resulting in aberrant gene expression [39] and the reproductive status of recipients carrying the pregnancy [40]. There was no pregnancy loss after Day 45 and viable foals were born in each group, demonstrating that the pre-IVM treatment with cAMP modulators did not have an adverse effect on fetal development. Similarly, in mice, Albuz et al. [10] showed that viable offspring can be obtained following the transfer of embryos produced from oocytes subjected to the SPOM system.

The success of nuclear transfer depends on the ability of the donor cells to be reprogrammed to a totipotent state, guided by the reprogramming factors present in the recipient cytoplast [41]. The developmental plasticity of cells from different lines is an important factor that determines the capacity of embryos to develop and give rise to healthy offspring [42,43]. In this study, pregnancies were established, and healthy cloned foals were obtained from all the cell lines used to produce the transferred blastocysts, demonstrating that complete cellular reprogramming to a totipotent state was achieved. Overall, viable foals were obtained from 17.4% (4/23) of the blastocysts transferred, which equates to approximately six embryo transfers for each foal born. This foaling rate is comparable to that reported previously for SCNT embryos [19].

The relationship between embryonic morphological features and pregnancy outcomes was also evaluated. Day 7 blastocysts classified as being of Grade 1 or 2 quality, according to well-defined criteria [21], were transferred to recipient mares. The morphological properties of in vitro-produced (IVP) equine blastocysts have been associated with their speed of development, and embryos that develop faster are more likely to be classified as Grade 1 and have a greater chance of generating births [44,45,46]. In the present study, births were only obtained from Grade 1 embryos. Assessing the kinetics of embryonic development is now routine practice to predict the outcome of transferred embryos in domestic species and humans [47].

Regarding the recipient mare’s day post-ovulation, similar pregnancy outcomes were achieved when ET was carried out 4 or 5 days after ovulation. In retrospective studies where many more ICSI-produced embryos were transferred to recipient mares on days 3–6 after ovulation, the best pregnancy rates were obtained when ET was performed on Day 4 [44,48], suggesting that the mare’s uterine environment and stage of IVP embryo development are optimally synchronized on that day. The success of equine ET is influenced by multiple factors, including other recipient mare factors such as age and uterine tone [49,50]. Given the multitude of factors at play, the relatively small number of embryos transferred to recipient mares in the present study likely precluded the detection of any differences between groups.

## 5. Conclusions

In conclusion, the results show that pre-IVM treatment with cAMP modulators for 4 or 18 h did not enhance the quality of equine oocytes, as the rates of oocyte maturation, couplet fusion, embryonic cleavage, and development to the blastocyst stage did not differ significantly from those of the Control group. Indeed, the rates of blastocyst formation tended to be lower in the Pre-IVM 4 h group, compared with the Control group. Following the transfer of blastocysts to recipient mares, four cloned foals were generated, including three from embryos produced using oocytes treated with the cAMP modulators, demonstrating for the first time that this treatment is compatible with full-term development in horses. Furthermore, the four cloned foals were derived from four different cell lines, demonstrating the reliability of the SCNT methods used. While effects on pregnancy outcomes due to the various factors analyzed (pre-IVM treatment, donor cell line, embryo grade, and recipient mare’s day post-ovulation) were not detected, transferring greater numbers of SCNT embryos may reveal significant differences. Regardless, as the cAMP modulators exerted no beneficial effects under the pre-IVM conditions described here, the findings do not support the use of the so-called SPOM system for equine oocyte maturation, adding to the controversy in this area. Further studies are needed to evaluate the merit of biphasic IVM approaches that modulate cAMP levels during equine oocyte maturation.

## Figures and Tables

**Table 1:** Effect of cAMP-modulating pre-IVM treatments on pregnancy and foaling.

Group	Recipient Mares Total	Pregnant at Day 14 ^1^	Pregnant at Day 45 ^1^	Pregnant at Day 90	Mares Foaling
Control	4	1 (25.0%)	1 (25.0%)	1	1
Pre-IVM 4 h	2	1 (50.0%)	1 (50.0%)	1	1
Pre-IVM 18 h	17	8 (47.1%)	2 (11.8%)	2	2
Total	23	10 (43.5%)	4 (17.4%)	4	4

^1^ Values in parentheses are the percentage pregnant of total recipient mares.

**Table 2:** Effect of the donor cell lines on pregnancy and foaling.

Donor Cell Line	Recipient Mares Total	Pregnant at Day 14 ^1^	Pregnant at Day 45 ^1^	Pregnant at Day 90	Mares Foaling
#01	10	3 (30.0%)	1 (10.0%)	1	1
#02	5	1 (20.0%)	1 (20.0%)	1	1
#03	4	3 (75.0%)	1 (25.0%)	1	1
#05	4	3 (75.0%)	1 (25.0%)	1	1

^1^ Values in parentheses are the percentage pregnant of total recipient mares.

**Table 3:** Effect of embryo grade on pregnancy and foaling.

Embryo Grade	Recipient Mares Total	Pregnant at Day 14 ^1^	Pregnant at Day 45 ^1^	Pregnant at Day 90	Mares Foaling
Grade 1	16	8 (50.0%)	4 (25.0%)	4	4
Grade 2	7	2 (28.6%)	0 (0.0%)	0	0

^1^ Values in parentheses are the percentage pregnant of total recipient mares.

**Table 4:** Effect of the recipient mare’s day post-ovulation at embryo transfer on pregnancy and foaling.

Day Post- Ovulation	Recipient Mares Total	Pregnant at Day 14 ^1^	Pregnant at Day 45 ^1^	Pregnant at Day 90	Mares Foaling
Day 4	7	3 (42.9%)	2 (28.6%)	2	2
Day 5	16	7 (43.8%)	2 (12.5%)	2	2

^1^ Values in parentheses are the percentage pregnant of total recipient mares.

## Data Availability

The data that support the findings of this study are available from the corresponding authors upon reasonable request.
